# Nicht-Direktivität als Leitkategorie in der humangenetischen Beratung in zeithistorischer Betrachtung

**DOI:** 10.1515/medgen-2022-2148

**Published:** 2022-09-22

**Authors:** F. Söhner, V. Rolfes, W. Hofmann, K. Zerres, H. Fangerau, M. Krischel

**Affiliations:** Institut für Geschichte, Theorie und Ethik der Medizin Centre for Health and Society, Medizinische Fakultät Heinrich-Heine-Universität Düsseldorf, Moorenstraße 5, 40225 Düsseldorf, Deutschland; MVZ genetikum® GmbH. Genetische Beratung & Diagnostik, Lautenschlagerstr. 23, 70173 Stuttgart, Deutschland; Institut für Humangenetik und Genommedizin, Universitätsklinikum der RWTH Aachen, Pauwelstr. 30, 52074 Aachen, Deutschland

**Keywords:** Oral History, Zeitgeschichte, Humangenetische Beratung, Nicht-Direktivität, Autonomie, oral history, contemporary history, genetic counselling, non-directivity, autonomy

## Abstract

In diesem Beitrag beschreiben und analysieren wir auf der Basis von gedruckten Quellen und Oral History Interviews die Frage der Nicht-Direktivität in der humangenetischen Beratung in Deutschland im Zeitraum von 1970 bis 2010. Dabei steht insbesondere die Frage im Fokus, inwieweit die fachwissenschaftlichen und gesellschaftlichen Diskurse zu ethischen Fragen in der genetischen Beratung von Fachkundigen wahrgenommen und inwieweit Aspekte der nicht-direktiven Beratung diskutiert wurden. Wie die Ergebnisse zeigen, maßen Fachkundige nicht nur rückblickend der Autonomie von Ratsuchenden einen hohen Stellenwert bei und lehnten „direktive“ Beratungen zumindest in öffentlichen Positionierungen ab. Ethische Begründungsansätze betrachteten sie hier als zentral.

## Abstract (en)

In this article we describe and analyse the issue of non-directivity in human genetic counselling in Germany between 1970 and 2010 based on printed sources and oral history interviews. The focus is on the extent to which the ethical aspects in genetic counselling were debated among human geneticists and to what extent aspects of non-directivity were discussed.

As the results show, it was not only in retrospect that experts attributed great importance to the autonomy of those seeking advice and rejected “directive” advice, at least in public positions. They considered ethical justification to be central here.

## Ausgangspunkt und Rahmensetzung

In einem Positionspaper der Deutschen Gesellschaft für Humangenetik (GfH) aus dem Jahre 2007 werden drei primäre Ziele der medizinischen Genetik formuliert: Beratung, Diagnostik und Therapie. Darüber hinaus distanzierte sich die Gesellschaft von eugenischen Positionen [1]. Das Papier wurde vor dem Hintergrund einer ersten Beratung über das Gendiagnostikgesetz im deutschen Bundestag verabschiedet, in der Einigkeit darüber bestand, dass die Verabschiedung eines solchen Gesetzes unumgänglich sei [2]. Das Dokument der GfH illustriert, dass die Fachgesellschaft humangenetisches Wissen als normgeladen reflektierte und ethische Erwägungen in ihre Positionsbildung zum Gesetzgebungsverfahren einbezog. Diese fachinterne Reflexion über die Rolle humangenetischer Beratung in und für eine Gesellschaft hatte sich seit den 1970er Jahren im spannungsgeladenen Dreieck zwischen fortschreitender Institutionalisierung der Humangenetik als Fachdisziplin, der Ausweitung ihrer diagnostischen Möglichkeiten und Furcht vor einer direktiven Neoeugenik etabliert. [3]

Einen Startpunkt der Debatte im deutschsprachigen Raum bildet das im Jahre 1968 veröffentlichte Buch zur genetischen Familienberatung als Leitfaden für Studierende und die Ärzteschaft von Walter Fuhrmann und Friedrich Vogel. Fuhrmann und Vogel formulierten hier ein direktives Beratungskonzept und begründeten dieses mit dem Wesen des ärztlichen Vertrauensverhältnisses, in der „*der Arzt Verantwortung für den Patienten übernimmt*, *dessen Entscheidung beeinflusst und auch für diese Mitverantwortung trägt“*. [4]

Brisant mutete eine kritische Haltung gegenüber genetischen Analysen und Präventionsangeboten an, die mit paternalistischer Begründung Ratsuchenden eine passive Rolle zuwies. Kritische Stimmen vermuteten hinter dem ärztlichen Paternalismus ein Einfallstor für eine neue eugenische Bewegung. Unterstützt wurden solche Befürchtungen durch die Bestrebungen der Humangenetik, mit politischer Unterstützung genetische Beratungs- und Untersuchungsstellen in Westdeutschland zu etablieren. Paradigmatisch stand hier u. a. ein Symposium zu „Genetik und Gesellschaft“ in Marburg 1969 mit dem Leitthema *„Erbgesundheit und Leistungsfähigkeit künftiger Generationen“*. [5] Auf dieser Tagung diskutierten Fachkräfte ein Forschungsprogramm zur „Sozialgenetik“ wie auch ein Konzept für eine genetische Beratungsstelle, das 1972 mit der „Genetischen Beratungsstelle für Nordhessen“ umgesetzt wurde. [6] Die Politik ließ sich überzeugen und so hieß es im Gesundheitsbericht der Bundesregierung von 1970: „*Humangenetische Gesichtspunkte müssen künftig bei der Geschlechtserziehung, Familienplanung und Eheberatung mehr Berücksichtigung finden*“. [7]

War bis in die Mitte der 1960er Jahre genetische Beratung nur auf Anfrage von Seiten der Anthropologie oder Medizin mit unterschiedlichen Spezialisierungen in Kliniken, Privatpraxen und den neu gegründeten Abteilungen für Humangenetik möglich gewesen, etablierte sich nun ein Angebot von institutionalisierten Beratungsstellen. Damit gewannen Fragen, wie die humangenetische Beratung durchzuführen sei, zunehmend an Relevanz.

In den folgenden Jahren schienen sich Kritisierende und Befürwortende dieser Beratungsstellen zumindest in Teilen zu arrangieren. Statt einer staatlich umgesetzten Neoeugenik zeigten sich die humangenetischen Beratungskonzepte eher als individualisierte und autonomiebasierte Anlaufstellen. [8]

Der vorliegende Beitrag beschreibt und analysiert auf Basis von gedruckten Quellen und Oral History Interviews den Umgang mit ethischen Fragen in der humangenetischen Beratung v. a. aus der Binnenperspektive des Faches. Dazu werden die Diskussionen untersucht, die in der Bundesrepublik im Zeitraum von 1970 bis 2010 zu Wertkonflikten zur Beratungspraxis geführt wurden. Folgende drei Konzepte sollen dabei im Mittelpunt stehen, bildeten sie doch den Kulminationspunkt der Debatte: Respekt vor der Autonomie von Ratsuchenden, „informed consent“ und Nicht-Direktivität. [9]

## Methoden und Quellen

Der Beitrag geht von der Frage aus, inwiefern ethische Aspekte wie „informed consent“ und Nicht-Direktivität die Etablierung einer humangenetischen Beratungspraxis aus Sicht von Humangenetikerinnen und Humangenetikern begleitet haben. Der Fokus liegt auf dem Zeitraum zwischen den markanten fachlichen Zäsuren von 1970 (Publikation des von G. Gerhard Wendt herausgegebenen Tagungsbands „Genetik und Gesellschaft“) und 2010 (Gendiagnostikgesetz). Ausgehend von den Auswirkungen gesellschaftlicher Pluralisierungs- und Individualisierungsprozesse auch auf die Humangenetik [8] wird untersucht, inwieweit die fachwissenschaftlichen und gesellschaftlichen Diskurse zu ethischen Fragen in humangenetischen Beratungskonzepten von Fachkundigen wahrgenommen wurden und inwieweit Aspekte der Direktivität von Beratung diskutiert wurden. Erinnerungen von fachkundigen Zeitzeuginnen und Zeitzeugen werden mit über eine systematische Literaturrecherche erhobenen Schriftquellen, schwerpunktmäßig Leitlinien, Stellungnahmen, Positionspapieren und Empfehlungen der Deutschen Gesellschaft für Humangenetik e. V. (GfH) und des Berufsverbands Deutscher Humangenetiker e. V. (BVDH), kontrastiert.

Um die Innenperspektive von Fachpersonen abzubilden, wurde die Methode der Oral History eingesetzt. [10] Als empirische Grundlage dienten 29 leitfadenorientierte lebensgeschichtliche Interviews mit Fachpersonen aus der Humangenetik. Die Methode der Datenerhebung, Beschreibung der Interviewpartner*innen und des Interviewleitfadens wurden bereits in vorhergehenden Ausgaben der „medizinischen genetik“ ausführlich dargestellt [10], [11], [12]. Demnach basiert der vorliegende Beitrag auf der standardisierten Methodik der qualitativen Sozialforschung nach Mayring. [13] Die Auswertung der leitfadengestützten Interviews erfolgte deduktiv und wurde nach der Grounded Theory induktiv erweitert. [13], [14], [15]

## Leitlinien, Stellungnahmen, Positionspapiere und Empfehlungen

Anfang der 1990er Jahre begannen Fachkundige, Empfehlungen und Leitlinien zur humangenetischen Beratung zu entwickeln. Leitlinien haben nicht den Status von staatlichen Gesetzen, gelten aber als zentrale Instrumente der Qualitätsentwicklung im Gesundheitswesen mit dem wesentlichen Ziel, die medizinische Versorgung durch die Standardisierung nach aktuellem Wissensstand zu verbessern. Sie vermitteln auf Basis von Handlungsempfehlungen einen klaren Handlungs- und Entscheidungsraum für die in der Praxis Tätigen. [16], [17] Auch der Umgang mit ethischen Dilemmata in der humangenetischen Beratung wurde in fachlichen Erklärungen und Leitlinien festgelegt. [18]

Im Vergleich dreier Leitlinien der Fachgesellschaft aus den Jahren 1996, 2007 und 2011 [9], [19], [20] lässt sich die steigende Akzeptanz der nicht-direktiven Beratung nachzeichnen. Thematisiert werden v. a. folgende Aspekte: an der Beratung beteiligte Personen, Praktiken und Ablauf des genetischen Beratungsgesprächs, Informationsvermittlung, Werte und beraterische Grundhaltungen, Umgang mit Problemen, Interaktion mit anderen Anbietenden von Gesundheitsleistungen sowie zukünftige Entwicklungen. [21] Als Teil des fachlichen Wertekonzepts und persönlicher Haltung gehen diese auf unterschiedliche Weise auf die Konzepte Autonomie, „informed consent“ und Nicht-Direktivität ein: Es herrscht in allen Versionen der Leitlinie Konsens darüber, dass die Kommunikation im Sinne einer personenzentrierten Beratung zu erfolgen hat.

Obgleich bereits in Fachbeiträgen diskutiert, tritt die ausdrückliche Formulierung der Entscheidungsautonomie der Ratsuchenden in den ersten beiden Leitlinien von 1996 und 2007 noch nicht auf. Ausdrücklich auf das Konzept der Autonomie wird erstmals 2011 hingewiesen. Es wird darauf verwiesen, dass „*Entscheidungen des Patienten* […] *das Ergebnis des kommunikativen Prozesses der Genetischen Beratung* [darstellen und] *in der Regel vom Berater mitgetragen* [werden].“ Dennoch kann ein potentieller Dissens auf Grund der „*Autonomie von Patient und Berater*“ prinzipiell nicht ausgeschlossen werden. [9] Es wird dabei betont, dass die humangenetische Beratung ergebnissoffen sei und den Ratsuchenden helfen solle, „*medizinisch-genetische Fakten zu verstehen, Entscheidungsalternativen zu bedenken und so informierte, eigenständige und tragfähige Entscheidungen zu treffen*.“ [9] Dieser Anspruch an Nicht-Direktivität der genetischen Beratung findet sich anders als die Autonomie auch schon in den früheren Leitlinien.

Die Ausführlichkeit der Regelungen sowie die fortschreitenden Erweiterungen sind ein Indiz dafür, dass die genetische Beratung als wichtigstes Instrument für die individuelle Kommunikation von Wahrscheinlichkeiten gelten kann. Insbesondere die neueren beiden Leitlinien (2007, 2011) gehen in gesonderten Punkten auf den Aspekt der humangenetischen Stellungnahme ein.

Die aktuelle S2-Leitlinie zur humangenetischen Diagnostik und genetischen Beratung aus dem Jahr 2018 fällt zwar nicht mehr in den Untersuchungszeitraum dieser Veröffentlichung, es soll aber trotzdem erwähnt werden, dass der Trend sich hier fortsetzt. Hier heißt es knapp: „*Die Genetische Beratung zeichnet sich durch einen ergebnisoffenen, non-direktiven Ansatz aus*.“[18] Es wird auf zwei Publikationen von Wolff und Jung verwiesen. [22] Wolff und Jung veröffentlichten wegweisende Publikationen und Schulungsmaterial zu nicht-direktiver humangenetischer Beratung. [23], [24] Wolff war von 1993 bis 2005 Vorsitzender der Kommission für Grundpositionen und Ethische Fragen der Deutschen Gesellschaft für Humangenetik und bis 2018 Vorsitzender der Kommissionen Leitlinien Genetische Beratung und Qualitätssicherung, Christine Jung war von 2005 bis 2009 zweite Vorsitzende der Kommission für Grundpositionen und Ethische Fragen, praktizierte als niedergelassene Humangenetikerin in Karlsruhe und Mannheim und ist seit 2020 an der Medizinischen Universität Innsbruck als Dozentin im Masterstudiengang Genetisches und Genomisches Counselling tätig. In ihrem Vorschlag für ein Konzept der Weiterbildung in ethischen und psychologischen Grundlagen genetischer Beratung schlugen Wolff und Jung vor, „*Nichtdirektivität als Beratungskonzept zugunsten des Begriffs der Erfahrungsorientiertheit als Beratungsgrundhaltung aufzugeben*.“ [15] Sie begründen dies, da der Anspruch von Nichtdirektivität der Beratung vorgeben würde, inhaltlichen Einfluss vermeiden zu können, wohingegen der Ansatz der Erfahrungsorientiertheit versuchen würde zu einem „*bewussteren Umgang mit dem manipulativen Potential von Beratung zu kommen*.“ [23]

Auch aus Empfehlungen und Positionspapieren lässt sich eine zunehmende Akzeptanz der nicht-direktiven Beratung seit den 1990er Jahren ablesen. Bereits 1996 hatten die Vorstandsmitglieder von GfH und Berufsverband Deutscher Humangenetiker (BVDH) festgestellt, dass Kenntnisse der ethischen Grundlagen ein unerlässlicher Teil der Weiterbildung im Gebiet Humangenetik seien. In diesem Kontext wiesen sie ausdrücklich auf das von Wolff und Jung 1996 vorgestellte Konzept hin und schätzten es als quantitativ und qualitativ empfehlenswert ein. [25] Ein Positionspapier der GfH aus dem Jahr 2007 fordert die „*aktive Förderung von individueller Autonomie und Entscheidungsfreiheit“* im Bereich der Lebens- und Familienplanung, die „*aktiv vor den Interessen Dritter oder privater und öffentlicher Institutionen geschützt*“ werden solle. Im Bereich der humangenetischen Beratung wird die Nicht-Direktivität als „*allgemein akzeptiert*“ charakterisiert. [26] Noch deutlicher heißt es in einem weiteren Positionspapier zu den „*Kernkompetenzen des Fachgebiets Humangenetik in der heutigen medizinischen Versorg*ung“ aus dem Jahr 2015: „*genetische Beratung erfolgt grundsätzlich non-direktiv*“. [27]

Eine jüngst erschienene Untersuchung zum Thema „Nicht-Direktivität und Partizipative Entscheidungsfindung in der genetischen Beratung über Pränataldiagnostik“, in der anhand einer Literaturrecherche 38 internationale Studien qualitativ verglichen wurden, entwickelt diese Frage weiter und zeigt auf, dass „*weder die Nicht-Direktivität noch die Partizipative Entscheidungsfindung als starre Konzepte einer Beratung umsetzbar sind, sondern entsprechend der unterschiedlichen Phasen eines Beratungsgespräches besondere Beachtung finden*.“ [28] Diese Betrachtungen verweisen auf die partizipative Entscheidungsfindung als passendes Kommunikationsmodell, das Beratenden in informativen Beratungsphasen ein individuell auf die Ratsuchenden zugeschnittenes Gespräch ermögliche und sowohl Entscheidungsfindung als auch Zufriedenheit der Ratsuchenden verbessere. [15], [28] Die partizipative Entscheidungsfindung wird verstanden als fortwährender Prozess, in dem neben den Ratsuchenden verschiedene betreuende Berufsgruppen sowie Angehörige einzubeziehen sind. Als zentral für deren Gelingen wird gesehen, das Gespräch den Kompetenzen und Bedürfnissen der involvierten Personen anzupassen, adäquate Hilfestellungen und verständliche Informationen anzubieten und auch sicherzustellen, dass die Ratsuchenden die Informationen verstanden haben und für sich nutzen können. [29] In sensiblen Beratungsphasen gelte das Prinzip der Nicht-Direktivität als Prinzip der Wahl. [28]

## Oral History Interviews

In den 29 ausgewerteten Interviews äußerten sich 27 befragte Personen zu „ethischen Aspekten“ ihrer Arbeit. In 12 der Interviews wurden konkret ethische Aspekte der humangenetischen Beratung angesprochen. Die relevanten Interviewsequenzen lassen sich zwei Kategorien zuordnen: **(1)** Relevanz von Ethik für die professionelle Identität als Humangenetikerin oder Humangenetiker in den Aspekten Lehre, Kommunikation mit der Öffentlichkeit und medizinischen Versorgung sowie **(2)** der Wandel hin zur nicht-direktiven genetischen Beratung ab etwa 1990, der in vielen Fällen ausdrücklich als Stärkung der Autonomie und damit (medizin-)ethisch geboten erinnert wurde.

Da alle Befragten als Hochschullehrende wirkten, war ihr Verständnis von Ethik als Teil professionellen Handelns auch davon bestimmt, in der Lehre und der Öffentlichkeit (teils kontrovers aufgeworfene) ethische Fragen zu beantworten. Eine Fachkraft berichtet:

*„Ethische Fragestellungen **haben eine ganz elementare Bedeutung***1Hervorhebung durch die Autorinnen und Autoren.. *Es hat überhaupt nie einen Schritt, gerade auch bei der genetischen Beratung, gegeben, bei der pränatalen Diagnostik schon gleich gar nicht, wo nicht ethische Überlegungen und Fragen ganz im Vordergrund standen*.“

[30]

Eine andere befragte Person erinnert sich:

*„Ja, die ethischen Fragestellungen der Humangenetik **waren natürlich ganz zentral***. [Wir] *sind als Institut ja angehalten gewesen, im Rahmen des Studium Generale Seminare zu halten*. […] *Die **Ethik spielt eine zentrale Rolle*****,** […] *in der Beratung ist das Grundlage*.“

[31]

Mehre Gesprächsbeteiligte berichteten, dass sie medizinethische Literatur rezipierten, um sich auf die universitäre Lehre und die genetische Beratung vorzubereiten.

In sieben der 29 ausgewerteten Interviews greifen die sich Erinnernden den Aspekt der „Nicht-Direktivität“ wörtlich auf, bei mehr als der Hälfte wurde er implizit oder explizit angesprochen. Besonders eindrücklich formuliert eine befragte Person:

*„In der sprechenden Humangenetik war* […] *das Schlüsselerlebnis die Überwindung der bis noch in die 80er Jahre hinein ganz stark fühlbaren eugenischen Orientierung der Beratung, also den Ratschlag Kinder zu kriegen oder nicht, und die **Hinwendung zur selbstverantwortlichen Entscheidungsfindung*** […] *natürlich, sehr, sehr stark gefördert von Helmut Baitsch*.“

[32]

Der Humangenetiker Helmut Baitsch war Mitbegründer des Sonderforschungsbereichs (SFB) 129 „Psychotherapeutische Prozesse“ (Ulm 1980). Die Arbeit des SFB trug dazu bei, dass Rollenspiel-Übungen zu Beratungssituationen vielerorts Bestandteil der medizinischen Ausbildung wurden. Der Einfluss dieses Forschungsprojekts und eine daraus resultierende Orientierung an den selbstverantwortlichen Entscheidungen der Ratsuchenden wurden von einzelnen Gesprächsbeteiligten gar als stark richtungsweisend wahrgenommen. Mehrere Personen betonten Baitschs Rolle bei der Etablierung der nicht-direktiven Beratung.

Andere Befragte erinnerten sich an die Formulierung eines „*Konzepts zur Weiterbildung in ethischen und psychologischen Grundlagen genetischer Beratung*“, das von Gerhard Wolff und Christine Jung [22], [23] 1996 vorgelegt wurde. Während einzelne Interviews darauf verweisen, dass bereits in den 1980er Jahren die Nicht-Direktivität als Richtschnur der Beratung gegolten habe, wird im folgenden Gespräch hervorgehoben, dass um die Durchsetzung dieses Konzepts gerungen werden musste:

textit„Ich wurde da damit konfrontiert automatisch in der genetischen Beratung […] *Wir haben die Probleme angesprochen mit unseren Ratsuchenden*. [Wir] ***haben verzweifelt gekämpft, nicht-direktiv** zu sein. Auch wenn* […] *am Schluss die Frage kam, Herr Doktor, was würden Sie denn machen? Da haben wir gesagt: Ja, schauen Sie, es ist Ihre Familie, Ihr Leben, Sie müssen das entscheiden*.“

[32]

Nur wenige Gesprächsbeteiligte verwendeten den Terminus des „informed consent“, doch mehr als die Hälfte der Befragten setzten sich mit diesem Aspekt auch ohne direkte Nachfrage kritisch und reflektiert auseinander.

Es liegt der Schluss nahe, dass eine nicht-direktive Beratung und damit die Achtung der Autonomie daran zu scheitern schien, dass manche Ratsuchende (*„weil* […] *die kognitiven Voraussetzungen nicht da waren*.“[32]) scheinbar nicht fähig waren, eine informierte Zustimmung zu erteilen. Hier zeigt sich ein enger Zusammenhang der genannten relevanten ethischen Aspekte, die als zentral für die Beschreibung und Analyse der lebensgeschichtlichen Interviews gelten können.

Der inhaltsanalytische Vergleich der professionellen Leitlinien zeigt, dass diese den Aspekt behandeln, sich ihm jedoch unterschiedlich begrifflich und strukturell widmen. Insgesamt lässt sich von Version zu Version eine stärkere Aufmerksamkeit auf die „informierte Zustimmung“ beobachten.

In Bezug auf den Aspekt der „Nicht-Direktivität“ zeichnet sich in den individuellen Äußerungen eine mehrheitliche Einstellung ab, Ratsuchende in einer selbstverantwortlichen Entscheidung zu unterstützen. Auch wenn in diesem Zusammenhang das aktive Verwenden des Terminus „Nicht-/Non-Direktivität“ weniger häufig auftrat, bezog sich die Mehrheit der Befragten auf diesen professionellen Selbst-Anspruch. Auch die inhaltliche Analyse der Leitlinien zeigt, dass die Frage der Beeinflussung von Entscheidung durchaus eine Rolle spielte.

## Diskussion und Ausblick

Die Analyse der Interviews zeigt, dass in den Erinnerungen die Wahrnehmung der Bedeutung ethischer Fragen in der humangenetischen Praxis durchaus Raum findet. Auch wenn nicht direkt nach Standards humangenetischer Beratung gefragt wurde, verwiesen einzelne Gesprächsbeteiligte auf eine hohe Relevanz ethischer Aspekte für ihre beratende Praxis, manche äußerten die Wahrnehmung, dass sich daraus ergebende neue Aufgabenbereiche auf die professionelle Zusammensetzung in den Beratungseinrichtungen auswirkten. Am deutlichsten wurde der Einfluss ethischer Aspekte im Zusammenhang der beruflichen Qualifikation formuliert; dieser Faktor wurde in der Aus- und Fortbildung als wirkmächtig erinnert.

Einzelne Interviewsequenzen lassen den Schluss zu, dass die Frage der „Nicht-Direktivität“ von den sich Erinnernden seinerzeit reflektiert wurde, andere zeigen eher weniger deutlich, ob nicht-direktive Kommunikationsmodelle bereits vor der fachlichen Diskussion bewusst wahrgenommen wurden. Auch zeichnet sich ab, dass die Kategorie Direktivität – Nicht-Direktivität eher weniger polar, sondern vielmehr ineinander übergehend verstanden werden kann. Hier stellt sich die Frage, inwiefern ein einheitliches Verständnis nicht-direktiver wie partizipativer Gesprächsführungskonzepte bei Beratenden seinerzeit vorherrschte und bei den befragten Personen heute vorliegt.

Weiter deuten die Interviewsequenzen auf eine lebendige, evtl. auch ambivalente Diskussion im Vorfeld der Formulierung der Leitlinien. Auch die wörtlichen Stellungnahmen und Positionierungen in der Weiterentwicklung der Leitlinienversionen legen diesen Schluss nahe.

Die Analyse der Interviews zeigt, dass die Autonomie der Ratsuchenden als wesentlich erinnert wurde und darauf verwiesen wurde, dass eine „direktive“ Beratung von der Mehrheit des Faches abgelehnt worden sei.

In der Bewertung dieser Aussagen sollte bedacht werden, dass sich das ärztliche Vertrauensverhältnis in den letzten Jahrzehnten in der Humangenetik wie in der gesamten modernen Medizin grundlegend gewandelt hat und der Aspekt der Autonomie der Ratsuchenden weltweit an Bedeutung gewonnen hat. [33] Da Einspeichern und Abrufen von Erinnerungen zustands- und kontextabhängig sind, ist in diesem Zusammenhang zu diskutieren, inwiefern es sich bei den wiedergegebenen Erinnerungen um sich über die Jahre herausgebildete Narrative handelt. Dies bedeutet, dass Erinnerungen rekonstruiert werden auf Grund von Erfahrungen (zu Erwartungshaltungen, Diskursen), Wissen (zu Forschungsstand, Leitlinien) sowie emotionaler Konnotation. Damit einher gehen können einerseits Fehlerinnerungen, andererseits situationsabhängige Gewichtungen. Gleichzeitig ist hier zu fragen, ob und inwiefern die Befragten in den Gesprächen nicht die Leitlinien repetieren und inwieweit deren wiedergegebene Erinnerungen durch das Wissen um die derzeitige Akzeptanz seinerzeit polarisierender Aspekte beeinflusst werden.

In Bezug auf die forschungsleitende Frage, kann aus den schriftlichen Quellen geschlossen werden, dass ethische Fragen in der genetischen Beratung von Fachkundigen bereits früh wahrgenommen wurden und vor dem Hintergrund des gesellschaftlich-medialen Diskurses eine zunehmende Rolle spielten. Insbesondere die Aspekte des „informed consent“ wie der „Nicht-Direktivität“ wurden und werden in den Leitlinien behandelt. Die Betrachtung der Leitlinien und des fachlichen Diskurses machen deutlich, dass die Frage der Direktivität bereits früh diskutiert wurde. Ab den 1990er Jahren setzte sich das Paradigma der nicht-direktiven Beratung in Deutschland durch. Die Veröffentlichungen von Gerhard Wolff und Christine Jung haben dazu entscheidend beigetragen.

Tatsächlich findet sich hier eine deutliche Übereinstimmung der schriftlichen Überlieferung mit den mündlichen Äußerungen der Gesprächsbeteiligten. Hier treten ethische Fragestellungen als ein Aspekt auf, der für das Fach Humangenetik und die genetische Beratungspraxis prägend war. Es wurde die hohe Relevanz von Ethik für die professionelle Identität als Humangenetikerinnen und Humangenetiker in den Aspekten Lehre, Kommunikation mit der Öffentlichkeit und medizinischen Versorgung erkennbar.

Sowohl schriftliche Quellen als auch die erfassten Erinnerungen verweisen darauf, dass ethische Fragen der genetischen Beratung im Untersuchungszeitraum „natürlich ganz zentral“ waren.

Vor diesem Hintergrund ist die Frage zu stellen, wie das Verhältnis der beiden Quellengattungen (mündlich vs. schriftlich) zu bewerten ist. Auch ist davon auszugehen, dass die Kenntnis um die Leitlinien und die Debatten individuelle Erinnerungen beeinflussen. In diesem Zusammenhang liegt der Schluss nahe, dass erinnerte Erfahrungen und Haltungen einzelner Fachkundiger die Genese der Leitlinien beeinflusste. Klären ließe sich diese Frage über eine historische Analyse kollegialer Korrespondenzen oder fachlicher Debatten im Umfeld der Verabschiedung der Leitlinien.

Zusammenfassend betrachtet wird unabhängig von dieser Frage erkennbar, dass die professionellen Leitlinien zur humangenetischen Beratungspraxis nicht nur nach innen die Anforderungen an Qualität humangenetischer Dienstleistungen beschreiben, sondern auch nach außen die professionelle Auseinandersetzung mit spezifischen Fragestellungen dokumentieren. [34]
